# Vitamin D supplementation and its impact on leptin and interleukin-6 in women following religious intermittent fasting: a controlled study

**DOI:** 10.3389/fendo.2025.1700844

**Published:** 2025-11-26

**Authors:** Spyridon N. Karras, Konstantinos Michalakis, Maria Kypraiou, Fatme Al Anouti, Hana Fakhoury, Cedric Annweiler, Stefan Pilz, Marios Anemoulis, Antonios Vlastos, Costas Haitoglou, Uwe Riedmann, Neoklis Georgopoulos, Evangelos G. Papanikolaou, Georgios Tzimagiorgis

**Affiliations:** 1 Laboratory of Biological Chemistry, Medical School, Aristotle University, Thessaloniki, Greece; 2 Endocrine Practice, Department of Obesity and Metabolism, Athens, Greece; 3 Assisting Nature Centre of Reproduction and Genetics, Thessaloniki, Greece; 4 College of Natural and Health Sciences, Zayed University, Abu Dhabi, United Arab Emirates; 5 Department of Biochemistry and Molecular Medicine, College of Medicine, Alfaisal University, Riyadh, Saudi Arabia; 6 UNIV ANGERS, Health Faculty, University of Angers, Angers, France; 7 Department of Geriatric Medicine and Memory Clinic, Research Center on Autonomy and Longevity, University Hospital, Angers, France; 8 UNIV ANGERS, University of Angers, Angers, France; 9 Gérontopôle Autonomie Longévité des Pays de la Loire, Angers, France; 10 Robarts Research Institute, Department of Medical Biophysics, Schulich School of Medicine and Dentistry, the University of Western Ontario, London, ON, Canada; 11 Department of Internal Medicine, Division of Endocrinology and Diabetology, Medical University of Graz, Graz, Austria; 12 Division of Endocrinology, Department of Internal Medicine, Medical School, School of Health Sciences, University of Patras, Patras, Greece

**Keywords:** vitamin D, IL-6, leptin, supplementation, intermittent fasting, orthodox nuns

## Abstract

**Background:**

Vitamin D deficiency is highly prevalent in populations following intermittent or religious fasting, particularly Orthodox nuns with limited sun exposure and dietary restrictions. Vitamin D may modulate adipokines and inflammatory markers, but clinical evidence remains inconsistent.

**Objective:**

To investigate the effects of vitamin D supplementation on interleukin-6 (IL-6) and leptin concentrations in Orthodox nuns compared with non-supplemented controls.

**Methods:**

In this controlled, non-randomized trial, 33 Orthodox nuns received oral vitamin D_3_ supplementation (2,500 IU/day) for 16 weeks, while 42 age-matched women following Orthodox intermittent fasting served as controls. Anthropometric indices and serum concentrations of 25-hydroxyvitamin D [25(OH)D], IL-6, leptin, parathyroid hormone, insulin, and C-reactive protein were measured at baseline and follow-up.

**Results:**

At baseline, serum 25(OH)D concentrations were lower in the supplementation group compared with controls (23.4 ± 10.1 *vs*. 27.7 ± 11.2 ng/mL, *p* = 0.043). After 16 weeks, 25(OH)D increased significantly in the supplementation group (23.4 ± 10.1 *vs*. 33.9 ± 11.0 ng/mL, *p* < 0.001), with no change in controls (27.7 ± 11.2 *vs*. 28.5 ± 12.2 ng/mL, *p* = 0.941). Leptin showed a non-significant decrease in the supplementation group (24.4 ± 18.3 *vs*. 21.9 ± 13.5 ng/mL, *p* = 0.215), whereas in the controls, it remained unchanged (23.21 ± 14.67 *vs*. 24.05 ± 13.89 pg/mL, *p* = 0.365). IL-6 did not change significantly in either group. Exploratory multivariable regression did not reveal significant predictors of leptin changes.

**Conclusions:**

Vitamin D supplementation effectively corrected deficiency but did not significantly affect leptin or IL-6 concentrations. These results highlight the need for larger and longer studies to further clarify the immunometabolic impact of vitamin D supplementation in women practicing intermittent fasting.

## Introduction

Vitamin D is a secosteroid hormone with a central role in calcium–phosphate homeostasis, skeletal integrity, and multiple extraskeletal processes, including immune regulation, metabolic balance, and cardiovascular health (1, 2). Serum 25-hydroxyvitamin D [25(OH)D] is the most reliable biomarker of vitamin D status. Low concentrations of 25(OH)D are highly prevalent worldwide and have been linked to increased risks of metabolic syndrome, autoimmune disorders, and chronic inflammatory states (3–5). Populations with restricted sun exposure, such as women living in religious communities, are particularly prone to vitamin D deficiency due to conservative clothing habits and limited outdoor activities (6, 7).

Beyond its classical endocrine functions, vitamin D is increasingly recognized for its immunomodulatory properties. Experimental studies demonstrate that the active metabolite, 1,25-dihydroxyvitamin D, regulates T-cell differentiation, suppresses pro-inflammatory cytokine production, and enhances anti-inflammatory pathways (8, 9). Interleukin-6 (IL-6) is a key pro-inflammatory cytokine implicated in obesity, insulin resistance, and atherosclerosis, while leptin is an adipokine secreted by adipose tissue that bridges energy homeostasis with immune function (10, 11). Elevated IL-6 and leptin concentrations have been associated with systemic inflammation, cardiometabolic disease, and increased all-cause mortality (12, 13). Thus, the potential ability of vitamin D to modulate IL-6 and leptin levels has emerged as an attractive hypothesis for nutritional and therapeutic intervention.

Human trials investigating the effect of vitamin D supplementation on IL-6 and leptin have yielded inconsistent findings. Some randomized controlled trials (RCTs) reported reductions in circulating IL-6 or leptin following vitamin D repletion (14, 15), whereas others found no significant changes (16–18). Differences in baseline vitamin D status, supplementation dose and duration, and study population characteristics may account for these discrepancies. In particular, data are scarce in populations with a high risk for developing hypovitaminosis D, such as Orthodox monastic communities and lay people following intermittent or religious fasting practices, due to periodic abstinence from animal-derived foods and reduced intake of vitamin D-rich sources such as dairy and fish (19). In addition, coupled with limited sun exposure due to sartorial habits among Orthodox nuns, a particularly high prevalence of vitamin D deficiency has been reported previously in these populations (7, 20, 21).

The present study aimed to investigate the effects of 16-week vitamin D supplementation on IL-6 and leptin concentrations in Orthodox nuns compared with lay women from the general population following Orthodox intermittent fasting. Secondary objectives included examining correlations between changes in 25(OH)D and changes in IL-6 or leptin, as well as evaluating whether vitamin D repletion independently predicted these outcomes after adjusting for anthropometric and biochemical covariates.

## Methods

### Design

This was a controlled, non-randomized intervention study conducted between January and April 2025. Participants were divided into two groups: a supplementation group of 33 Orthodox nuns receiving a daily oral regimen of 2,500 IU vitamin D**_3_** (in the form of oil-based drops or softgel tablets) and a control group of 42 women following fasting practices without intervention.

### Study populations—interventions and follow-up

We included convenient samples of Orthodox nuns from two monastic communities and an age-matched cohort of adult lay women from the same region, recruited from local parishes and community networks in northern Greece. Participants were initially screened for eligibility through medical history, fasting blood sampling, and anthropometric assessment and were divided into two groups: a supplementation group (Orthodox nuns) receiving a daily oral regimen of 2,500 international units (IU) vitamin D_3_ daily (in the form of oil-based drops or softgel tablets) and a control group without intervention. Each monastic community followed a uniform supplementation form (either tablets or drops). Both groups followed the Athonian type of fasting as previously described (7, 19–24). The Orthodox nun group adopted an 8-h eating interval (08:00 to 16:00), as dictated by monastery dietary rules, while the control group consumed food from 09:00 to 17:00. The inclusion criteria were age between 30 and 50 years and regular participation in fasting practices for lay women. The exclusion criteria were current vitamin D supplementation, body mass index (BMI) ≤25, amenorrhea ≥3 months, pregnancy, administration of medications that can alter weight, glucose and lipid metabolism, and chronic kidney or liver disease. Participants in the control group were age-matched lay women recruited from local parishes within the same region and time frame as the monastic cohort to minimize seasonal and environmental variability, particularly in terms of sunlight exposure. Although physical activity levels were not objectively quantified, both groups shared similar daily schedules with low-intensity activities and limited outdoor exposure.

Adherence to dietary plans was evaluated with a 3-day food record (two weekdays and one weekend day) at the end of the study period, while the Nutrition Analysis Software Food Processor [https://esha.com/products/food-processor/ (accessed on 2 August 2025)] was used to analyze food records. A follow-up evaluation was conducted 16 weeks after supplementation initiation for the intervention group. The control group underwent assessments on the same timeline without receiving supplementation.

### Anthropometric measurements and biochemical analysis

Body weight and height were recorded. Body weight was measured to the nearest 0.01 kg using a calibrated digital scale (K-Tron P1-SR, Onrion LLC, Bergenfield, NJ, USA), with participants barefoot and wearing light clothing. BMI was then computed by dividing weight (kg) by height squared (m^2^). Total and visceral body fat were assessed by bioelectrical impedance analysis (BIA) (SC-330 S, Tanita Corporation, Tokyo, Japan) (25, 26). Fasting blood samples were collected in the morning, and serum concentrations of [25(OH)D], intact PTH, insulin, interleukin-6, and leptin were measured at baseline and after 16 weeks in both groups. Blood was collected in EDTA and standard red top vacutainer tubes, allowed to clot for 30 min at room temperature, and centrifuged at 3,000 rpm for 10 min, and aliquots were immediately stored at −80°C until analysis. Fasting blood samples were also analyzed for serum insulin (μIU/mL) concentrations using standard enzymatic and immunoassay methods, respectively. Measurements of parathyroid hormone (PTH), 25-hydroxyvitamin D [25(OH)D], and insulin were performed on the COBAS e 602 module via electrochemiluminescence (ECL) assay. The reference values and inter-/intra-assay coefficients of variation were as follows: 15–65 pg/mL of PTH (1.6–6.9 pmol/L; intra: 2.5%–3.4%; inter: 1.1%–2.0%), 25(OH)D ≥30 ng/mL (intra: 3.4%–13.1%; inter: 2.2%–6.8%), and 5–20 μIU/mL of insulin (CV: 3.2%–6.2%). Interleukin-6 was measured through the Abcam US Human IL-6 20R2 ELISA assay (range 7.8–500 pg/mL and sensitivity = 1.6 pg/mL), and leptin was measured through the Abcam US Human Leptin ELISA assay (sensitivity = 4.65 pg/mL; range = 15.63–1,000 pg/mL).

### Ethical considerations

Written informed consent for inclusion in the study was provided by the participants. The study protocol adhered to the principles set forth in the Declaration of Helsinki. Approval for the inclusion of the monastic cohort was obtained in writing from the central ecclesiastical authority following submission of the full study documentation 1 year prior to study initiation. The study protocol was approved by the Institutional Review Board of Aristotle University of Thessaloniki, Greece (Approval No. 25224).

### Statistical analysis

Statistical analyses were conducted using SPSS (version XX). Data are presented as mean ± standard deviation (SD). Paired *t*-tests were used to compare baseline *vs*. post-intervention values within each group. Independent *t*-tests were applied to compare changes (Δ = post − baseline) between groups. Normality of data distribution for all continuous variables was tested using the Shapiro–Wilk test. When normality assumptions were not met, non-parametric tests (Wilcoxon signed-rank and Mann–Whitney *U*) were applied, yielding results consistent with the parametric analyses.

Additionally, a two-way repeated-measures ANOVA (group × time) was performed to assess the interaction between supplementation and time effects on serum biomarkers. This analysis confirmed the direction and significance of findings obtained from paired and independent *t*-tests. Correlations between Δ25(OH)D and ΔIL-6 or ΔLeptin were examined using Pearson correlation coefficients with 95% confidence intervals (CIs). Multivariable linear regression models were performed to test whether Δ25(OH)D independently predicted ΔIL-6 or ΔLeptin, adjusting for age, body weight, body fat percentage, and baseline 25(OH)D levels. Statistical significance was set at *p <*0.05.

## Results

**Figure 1:**
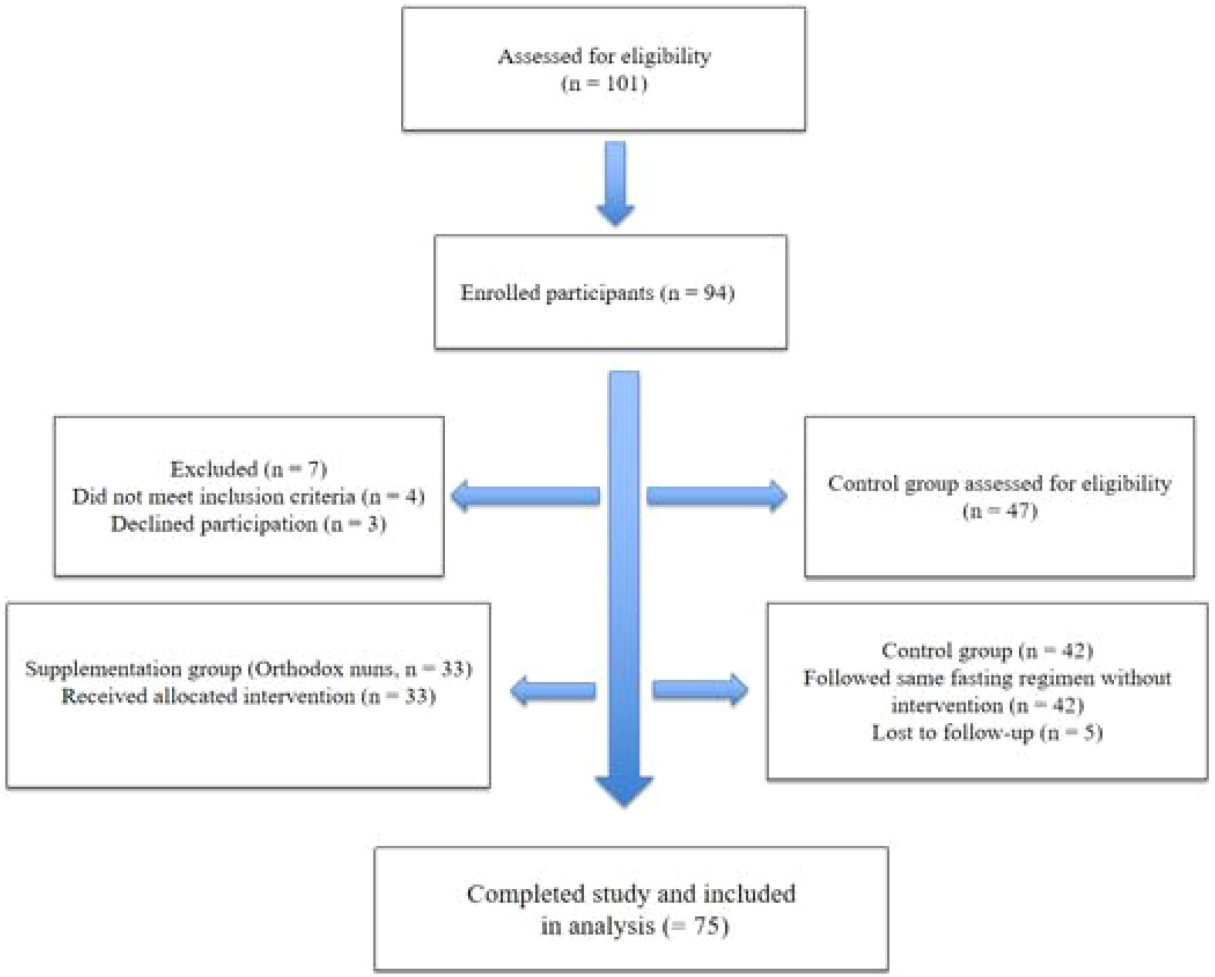
Flow diagram of participant recruitment, inclusion, allocation, and follow-up for both study groups.

A total of 101 women were initially assessed for eligibility. After screening and exclusions due to ineligibility or refusal to participate, 33 were allocated to the supplementation group and 42 to the control group. The flow of participant recruitment, inclusion, and follow-up for both groups is illustrated in **Figure 1**.

At baseline, the supplementation group (*n* = 33) and controls (*n* = 42) were comparable in anthropometric and biochemical characteristics, with the exception of serum 25(OH)D, which was significantly lower among nuns compared to controls (23.43 ± 10.08 *vs*. 27.66 ± 11.21 ng/mL, *p* = 0.043). Baseline IL-6 concentrations did not differ significantly between groups. Following the 16-week intervention, Orthodox nuns receiving vitamin D supplementation exhibited a robust increase in serum 25(OH)D concentrations, rising from 23.43 ± 10.08 to 33.93 ± 11.04 ng/mL (*p* < 0.001), while no change was observed in controls (27.66 ± 11.21 to 28.51 ± 12.21 ng/mL, *p* = 0.941). Leptin concentrations decreased numerically in the supplementation group (24.39 ± 18.28 to 21.93 ± 13.49 ng/mL), but the difference was not significant (*p* = 0.214). No significant changes were observed in the control group (23.21 ± 14.67 *vs*. 24.05 ± 13.89 pg/mL, *p* = 0.365). IL-6 and other metabolic parameters showed no significant differences within or between groups. Other parameters, including CRP, insulin, and muscle mass, did not show significant changes within or between groups. No solicited or unsolicited adverse events were reported in either group during follow-up, and no participant discontinued supplementation because of adverse effects. **Table 1** summarizes the baseline and post-intervention characteristics of both groups.

**Table 1:** Baseline and post-intervention values of anthropometric and biochemical parameters in the intervention and control groups.

Variable	Intervention baseline (*n* = 33)	Intervention post	*P*-value	Controls baseline (*n* = 42)	Controls post	*P*-value	*P*-value (between groups) post
Age (years)	42.4 ± 8.11			41.7 ± 7.91			0.771
BMI (kg/m²)	27.11 ± 3.21	26.7 ± 3.0	0.041	26.97 ± 3.17	27.09 ± 3.03	0.512	0.665
% Body fat	36.57 ± 4.82	35.8 ± 4.5	0.052	36.28 ± 4.73	36.11 ± 4.63	0.648	0.701
Muscle mass (kg)	44.20 ± 3.91	44.53 ± 4.01	0.327	44.19 ± 3.84	44.22 ± 3.98	0.822	0.732
CRP (mg/L)	2.11 ± 0.97	1.97 ± 0.85	0.081	2.08 ± 0.91	2.11 ± 0.97	0.611	0.612
25(OH)D (ng/mL)	23.43 ± 10.08	33.93 ± 11.04	<0.001	27.66 ± 11.21	28.51 ± 12.21	0.941	0.039
Insulin (µIU/mL)	9.13 ± 2.35	8.65 ± 2.15	0.067	9.04 ± 2.41	9.11 ± 2.32	0.932	0.841
Leptin (ng/mL)	24.39 ± 18.28	21.93 ± 13.49	0.214	23.21 ± 14.67	24.05 ± 13.89	0.365	0.561
IL-6 (pg/mL)	3.51 ± 1.46	3.40 ± 0.99	0.089	1.63 ± 0.77	1.62 ± 0.85	0.902	0.061

Values are presented as mean ± SD. *p*-values indicate within-group and between-group comparisons.

Pearson correlation analyses were performed to assess whether changes in vitamin D status were associated with those observed in IL-6, leptin, fat mass, insulin, and BMI. In the intervention group, no significant correlations were detected between Δ25(OH)D and ΔIL-6 (*r* = 0.158, 95% CI −0.208 to 0.485, *p* = 0.395) or ΔLeptin (*r* = 0.137, 95% CI −0.210 to 0.455, *p* = 0.438) (**Table 2**). In the control group, no significant correlations were observed (data not shown).

**Table 2:** Pearson correlations between changes in serum 25(OH)D [Δ25(OH)D] and IL-6, leptin, fat mass, insulin, and BMI.

Outcome	*r*	95% CI	*P*-value
ΔIL-6	0.16	(–0.21, 0.49)	0.40
ΔLeptin	0.14	(–0.21, 0.46)	0.44
ΔFat %	−0.18	(−0.62, 0.17)	0.31
ΔInsulin	−0.09	(−0.45, 0.28)	0.62
ΔBMI	−0.12	(−0.48, 0.25)	0.53

Values represent correlation coefficient (*r*), 95% confidence interval (CI), and *p*-value.

**Figure 2:**
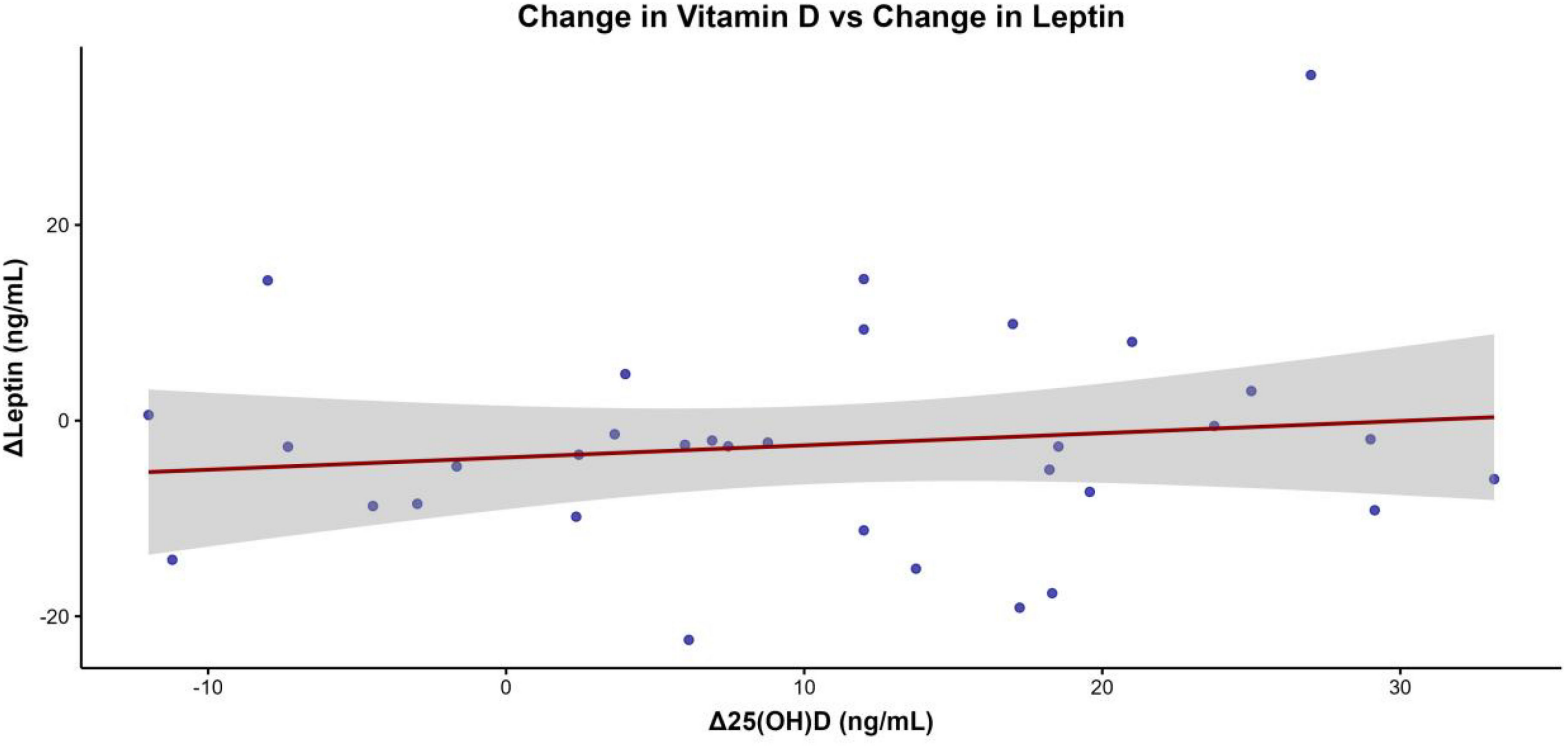
Scatterplot illustrating the association between changes in serum 25-hydroxyvitamin D [Δ25(OH)D] and changes in leptin concentrations (ΔLeptin) in the intervention group, with a regression line and its 95% confidence interval displayed.

The scatterplot of Δ25(OH)D vs. ΔLeptin in the intervention group (**Figure 2**) did not reveal any significant association.

In the control group, multivariable regression analyses did not reveal any significant predictors for changes in IL-6 or leptin (data not shown). Subgroup analysis by baseline 25(OH)D levels (<20 *vs*. ≥20 ng/mL) showed no significant differences in IL-6 and leptin concentrations in the intervention group.

## Discussion

The present study evaluated the effects of vitamin D supplementation on IL-6 and leptin concentrations in women practicing intermittent fasting, specifically Orthodox nuns compared with non-supplemented controls. A 16-week supplementation regimen effectively increased serum 25(OH)D concentrations in the intervention group.

Leptin concentrations showed a numerical decrease in the supplementation group, but this effect was not significant. IL-6 concentrations did not differ significantly within or between groups, and no associations were observed between Δ25(OH)D and changes in IL-6. The magnitude of the effect was small, and the absence of consistent correlations suggests that this association might be context-dependent, influenced by baseline adiposity, diet, and metabolic status.

Previous studies (27, 28) examining the relationship between vitamin D and leptin have yielded inconsistent results, with some reporting reductions, others increases, and several showing no effect. Meta-analyses also highlight this heterogeneity, which may be explained by differences in baseline vitamin D levels, degree of adiposity, and study design (29). The conflicting effect of vitamin D supplementation on leptin can be partly attributed to the variability of leptin in obesity, depending on the stage of weight change. In obese states, leptin levels are typically elevated due to leptin resistance, whereas after weight loss, leptin tends to normalize as sensitivity improves.

Our findings add to this debate by demonstrating neutral effects in a specific population of women adhering to intermittent fasting and religious dietary practices. This context may have amplified the response to supplementation, given the relatively low vitamin D concentrations at baseline and the potential interplay with body composition changes. The 16-week supplementation period was selected based on previous trials indicating that steady-state serum 25(OH)D concentrations are typically achieved within 8–12 weeks of daily dosing. Furthermore, studies have demonstrated that inflammatory and adipokine responses, including IL-6 modulation, can be detected within 12–16 weeks following vitamin D repletion (29).

Mechanistically, vitamin D might influence leptin secretion by modulating adipocyte gene expression, insulin sensitivity, and lipid metabolism. Experimental evidence shows that vitamin D can suppress leptin secretion in adipose tissue, while leptin itself may enhance vitamin D signaling by upregulating vitamin D receptor (VDR) expression. Leptin has been reported to regulate VDR expression in adipose tissue, immune cells, and osteoblasts, thereby enhancing the transcription of vitamin D-dependent genes. Conversely, vitamin D has been shown to regulate leptin gene expression in adipose tissue. Both leptin and vitamin D also exhibit anti-inflammatory properties, with implications for metabolic regulation, bone turnover, and immune health (29). These reciprocal regulatory pathways suggest that the relationship between vitamin D and leptin is bidirectional and complex, potentially depending on nutritional status, hormonal environment, and metabolic demands.

The particular dietary and lifestyle patterns of Orthodox nuns may also contribute to the observed effects (30). Intermittent fasting, modest caloric intake, and limited sun exposure are distinctive characteristics of this group, leading to both low baseline vitamin D levels and unique metabolic adaptations. Vitamin D supplementation in this context may have exerted a more pronounced effect on leptin compared to other populations. These results underscore the importance of considering cultural and lifestyle factors when interpreting the metabolic effects of vitamin D supplementation.

The clinical relevance of our findings lies in the observation that vitamin D supplementation, while effective in correcting deficiency, may not translate into broad improvements in metabolic or inflammatory markers among otherwise healthy women. Future research should include larger randomized trials with longer follow-up, higher supplementation doses, and stratification by baseline vitamin D status and inflammatory burden, in order to more precisely delineate the metabolic and immunomodulatory effects of vitamin D. The modest increase in serum 25(OH)D may relate both to the relatively conservative dose used (800–2,500 IU/day) and to individual variability in absorption efficiency. Factors such as dietary fat intake, gastrointestinal physiology, and the fasting pattern itself could have influenced bioavailability. Future studies using higher or personalized dosing regimens could help distinguish between dose insufficiency and absorption-related variability.

Several limitations should be acknowledged. First, the relatively modest sample size limits the statistical power to detect small-to-moderate effects and reduces the generalizability of the findings. Second, biomarkers such as leptin exhibit considerable intraindividual variability and may require repeated measures or larger cohorts for reliable assessment. Third, the non-randomized design may introduce bias, and the absence of a placebo control could have influenced participant expectations, although biomarker values remained stable in controls. Fourth, we did not assess seasonal variations, alternative dosage regimens, or potential interactions with dietary calcium intake. In addition, 24-h urine collections to evaluate hypercalciuria were not performed due to regulatory restrictions in the monastic community.

Moreover, the relatively small and demographically homogeneous sample, consisting exclusively of Orthodox women practicing religious fasting, limits the generalizability of our findings. These results may not directly extend to men or to individuals with different dietary, environmental, or lifestyle characteristics. Future studies including participants from diverse cultural and geographic backgrounds are warranted to confirm the external validity of these observations. Although physical activity levels were not objectively quantified, both groups shared similar daily schedules with low-intensity activities and limited outdoor exposure. The non-randomized design may therefore introduce residual confounding. Dietary intake was assessed through 3-day food records, which are inherently subject to recall and reporting bias. Despite the use of standardized guidance and validated nutrient analysis software, minor inaccuracies may have occurred, potentially diluting the strength of observed associations.

Only IL-6 was measured as a representative inflammatory cytokine due to resource and sample limitations. The inclusion of additional markers such as TNF-α, IL-1β, or high-sensitivity CRP would have provided a more comprehensive picture of systemic inflammation. Finally, the relatively short intervention period (16 weeks) may not have been sufficient to capture downstream or long-term effects of vitamin D supplementation.

The 16-week duration of supplementation may have been insufficient to capture the full extent or sustainability of vitamin D-related immunometabolic changes. Longer-term randomized controlled trials with extended follow-up are required to determine whether the observed neutral effects on IL-6 and leptin persist or evolve over time.

In conclusion, this controlled study demonstrated that daily vitamin D supplementation for 16 weeks effectively corrected deficiency among women practicing religious intermittent fasting but did not significantly alter leptin or IL-6 concentrations. The modest sample size, short duration, and limited inflammatory profiling warrant cautious interpretation. These findings highlight the need for larger, longer, and more mechanistically oriented studies to clarify the immunometabolic effects of vitamin D in populations with distinctive dietary and lifestyle patterns.

## Data Availability

The raw data supporting the conclusions of this article will be made available by the authors, without undue reservation.
