# Supplementary figures and images for: Vitamin D supplementation and its impact on leptin and interleukin-6 in women following religious intermittent fasting: a controlled study
Source: Front Endocrinol (Lausanne). 2025 Nov 26;16:1700844. doi: 10.3389/fendo.2025.1700844 (PMC12689337; doi:10.3389/fendo.2025.1700844)

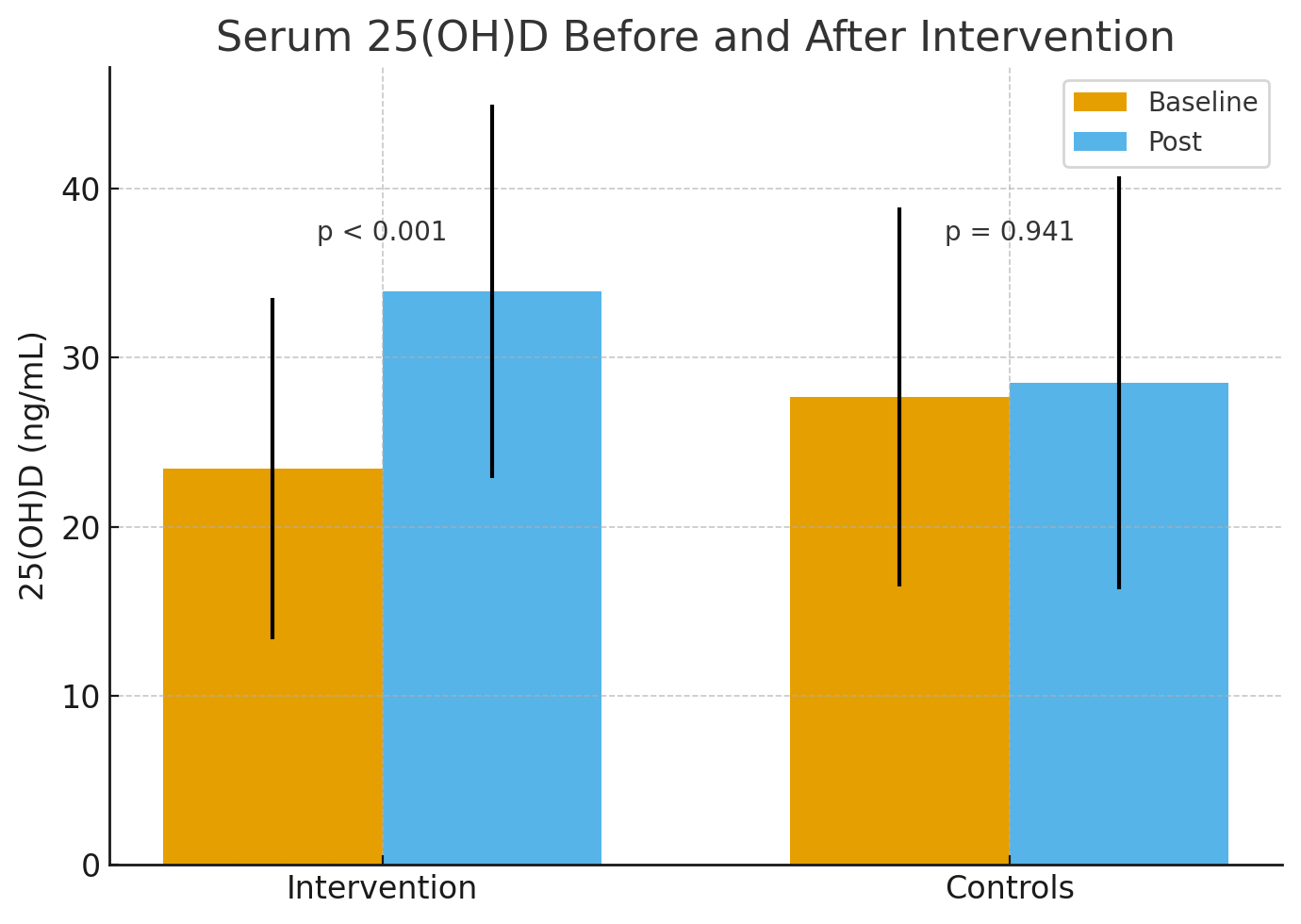

Supplement: Supplementary file 1 [file Image1.png]
